# Case of Irreducible Ileocecal Intussusception Due to Leiomyoma of the Colon

**DOI:** 10.7759/cureus.5583

**Published:** 2019-09-06

**Authors:** Amar R Patel, Avani R Patel, Harvey Rainville

**Affiliations:** 1 Internal Medicine, Northern California Kaiser Permanente, Fremont, USA; 2 General Surgery / Bariatric Surgery, Hackensack Meridian Health Mountainside Medical Center, Montclair, USA

**Keywords:** intussusception, ileoileal intussusception, leiomyoma, ileocecal valve, intestinal obstruction, irreducible ileocecal intussusception

## Abstract

Intussusception is defined as the telescoping of the proximal segment of the bowel into the distal segment. In most clinical cases, pediatric intussusception is much more common than adult intussusception. Pediatric intussusception is often due to viral or bacterial infections, which lead to the inflammation of lymphoid tissue in the intestine. Adult intussusception is typically secondary to tumors and idiopathic causes. Malignant tumors tend to affect the colon, while benign tumors affect the small intestine. Lipomas are the leading cause of benign tumors which cause intussusception. Conversely, adenocarcinomas are the leading cause of malignant tumors that cause intussusception.

Our case focuses on a young adult who developed intussusception secondary to a leiomyoma present near the ileocecal junction of the intestine. The treatment of intussusception caused by tumors is either surgical reduction or excision of the involved tissue. If excised, the specimens are sent to the pathology department for confirmation of the potential cause. If leiomyomas are suspected, staining is used to differentiate them from gastrointestinal stromal tumors (GISTs). These stromal tumors are unusual causes of intussusception, which require further research to determine their disease course and age at presentation.

## Introduction

The condition of intussusception is seen in the intestine. It was first described by Dr. Paul Barbette in 1674 and first treated by Sir Jonathan Hutchinson in 1871 [1]. Before 1871, intussusception was classified as a serious life-threatening condition that had a high mortality rate [1].

Intussusception occurs when the proximal segment of the intestine is invaginated in the distal segment. This would grossly appear very similar to a telescope being collapsed into a single segment. It is an acute condition and can occur in several different locations within the small or large bowel. Four different types of intussusception have been identified. They are known as ileo-colic, ileo-ileo-colic, colic-colic, and small bowel intussusception. Intussusception can be treated using enemas and surgery [1].

Ileocecal intussusception is typically a pediatric diagnosis and differs in etiology as compared to adults. Pediatric intussusception is most commonly caused by nonspecific causes such as viral or bacterial infections of the intestine. The infection will lead to the inflammation of the Peyer’s patches, which leads to the telescoping of the proximal segment of the ileum into the cecum. Compared to the pediatric population, the incidence of this condition is rarer in adults with the underlying cause usually a tumor [2].

The following case aims to present irreducible ileocecal intussusception due to leiomyoma of the colon.

## Case presentation

A 22-year-old female presented with intermittent abdominal pain for five days with additional complaints of vomiting and diarrhea. She had a past history of bipolar disorder and cholecystectomy secondary to gallstones (in 2011). The patient stated that her abdominal pain worsened progressively since her initial presentation to the hospital. The pain was present in the left upper abdominal quadrant and radiated towards the epigastrium and back. The pain was described as spastic and rated nine out of 10 in severity. It was aggravated by laying down flat and moving from side to side. The pain was relieved by vomiting and bowel movements. When asked, the patient described her bowel movements as ribbonlike, green in color, and without the presence of blood.

The patient first came into an urgent care facility with abdominal pain that was diagnosed as gastritis. Two days later, she came to the emergency department with abdominal pain that was progressively becoming worse. The patient did not have fever, cough, nausea, lightheadedness, headache, chest pain, or sick contacts. There was no significant travel history. For her obstetrics and gynecology history, she reported having a normal degree of cramps, rhythm, flow, and normal duration of menses. The patient’s abdominal examination demonstrated minimal left upper quadrant distension (greater than left lower and right upper quadrant), tenderness, and no guarding. No mass was palpated during the abdominal examination.

Laboratory investigations showed a hemoglobin (Hb) level of 9.7 grams per deciliter (g/dl), a hematocrit (HCT) of 35.9%, mean corpuscular volume (MCV) of 68 femtoliters (fL), and a red blood cell distribution width (RDW) of 16.1 fL. This is suggestive of iron deficiency anemia.

**Figure 1:**
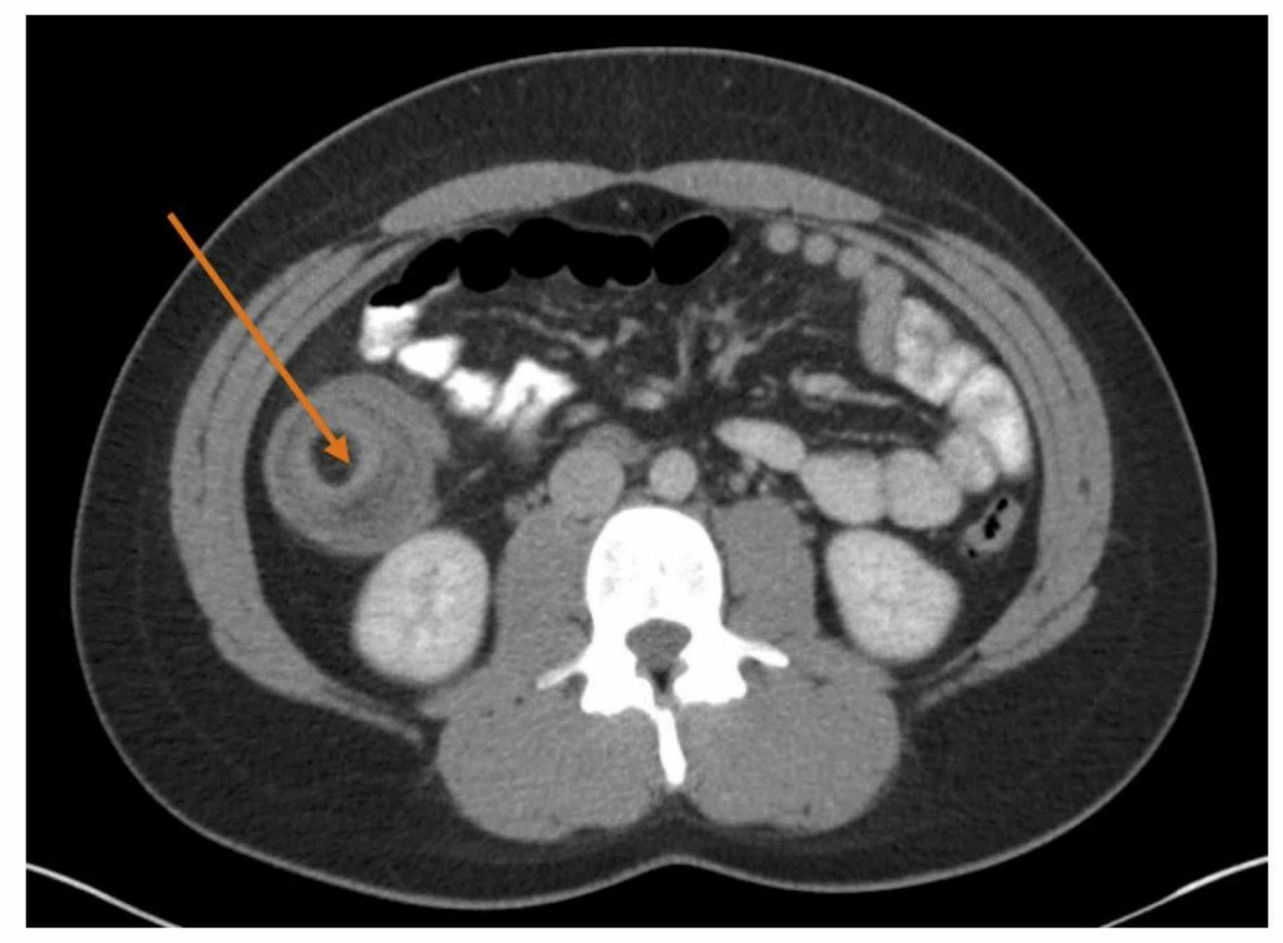
Computed tomography of abdomen and pelvis with contrast The transverse view of computed tomography (CT) of abdomen and pelvis with contrast. An ileocolic intussusception is present (orange arrow) with the terminal ileum telescoping into the ascending colon over an 8-centimeter interval.

**Figure 2:**
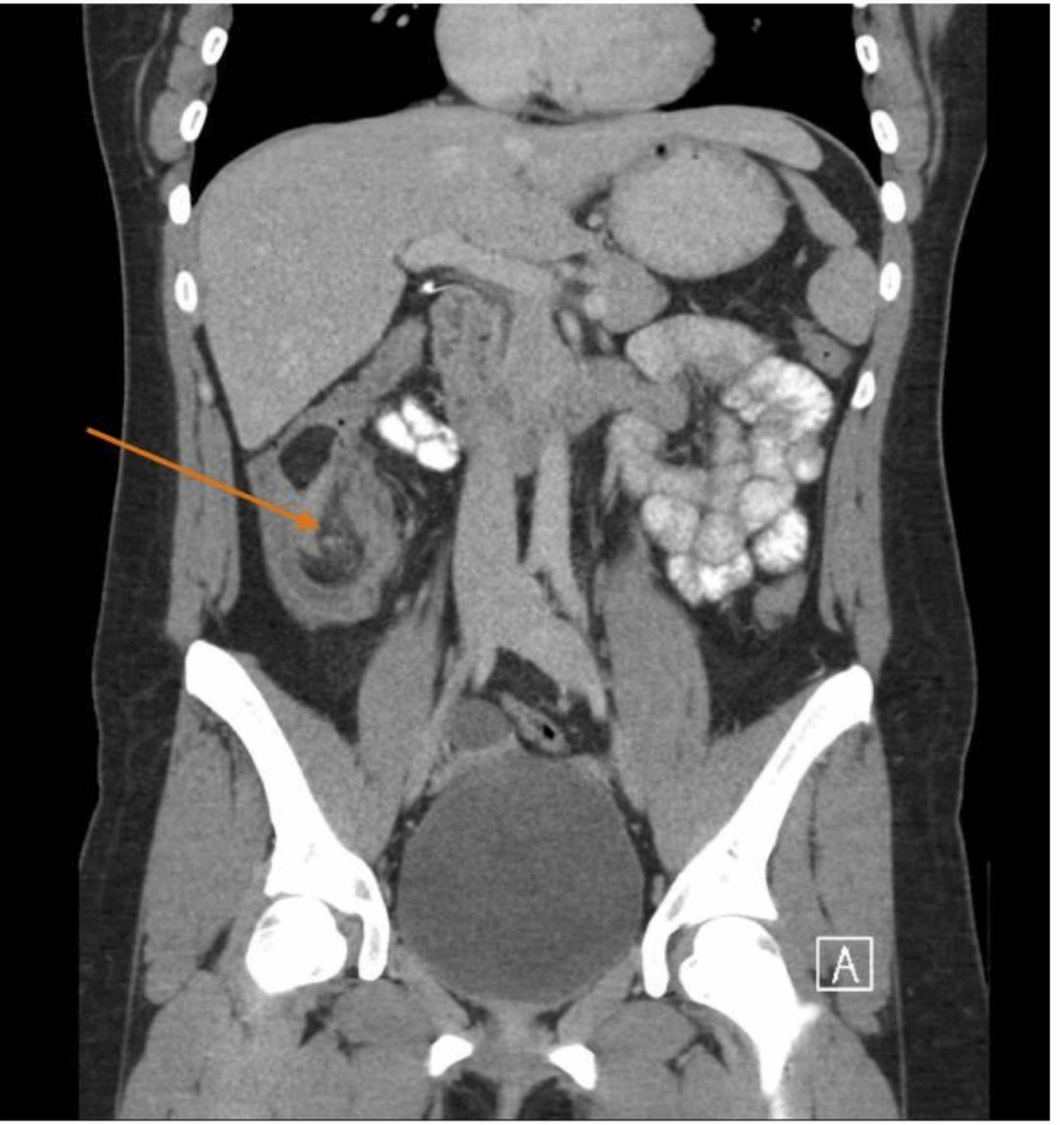
Computed tomography of abdomen and pelvis with contrast The axial view of a computed tomography scan of abdomen and pelvis with contrast. An ileocolic intussusception is present (orange arrow) with the terminal ileum telescoping into the ascending colon over an 8-cm interval.

A computed tomography (CT) scan of the abdomen and pelvis with contrast showed an ileocolic intussusception with the terminal ileum telescoping into the ascending colon over an 8-centimeter (cm) interval (see Figure 1 and Figure 2). A 2.9-cm round, fat, attenuated structure was seen within the lumen of the ascending colon in this region. This structure was suspicious for an intraluminal lipoma, which raised the possibility of a potential lead point mass. Moderate distension of the terminal ileum with fecalization (a nonspecific sign of bowel pathology) of the bowel contents was also noted. The patient was stabilized. Afterward, she was inserted with a nasogastric (NG) tube and placed on nil per oral (NPO).

**Figure 3:**
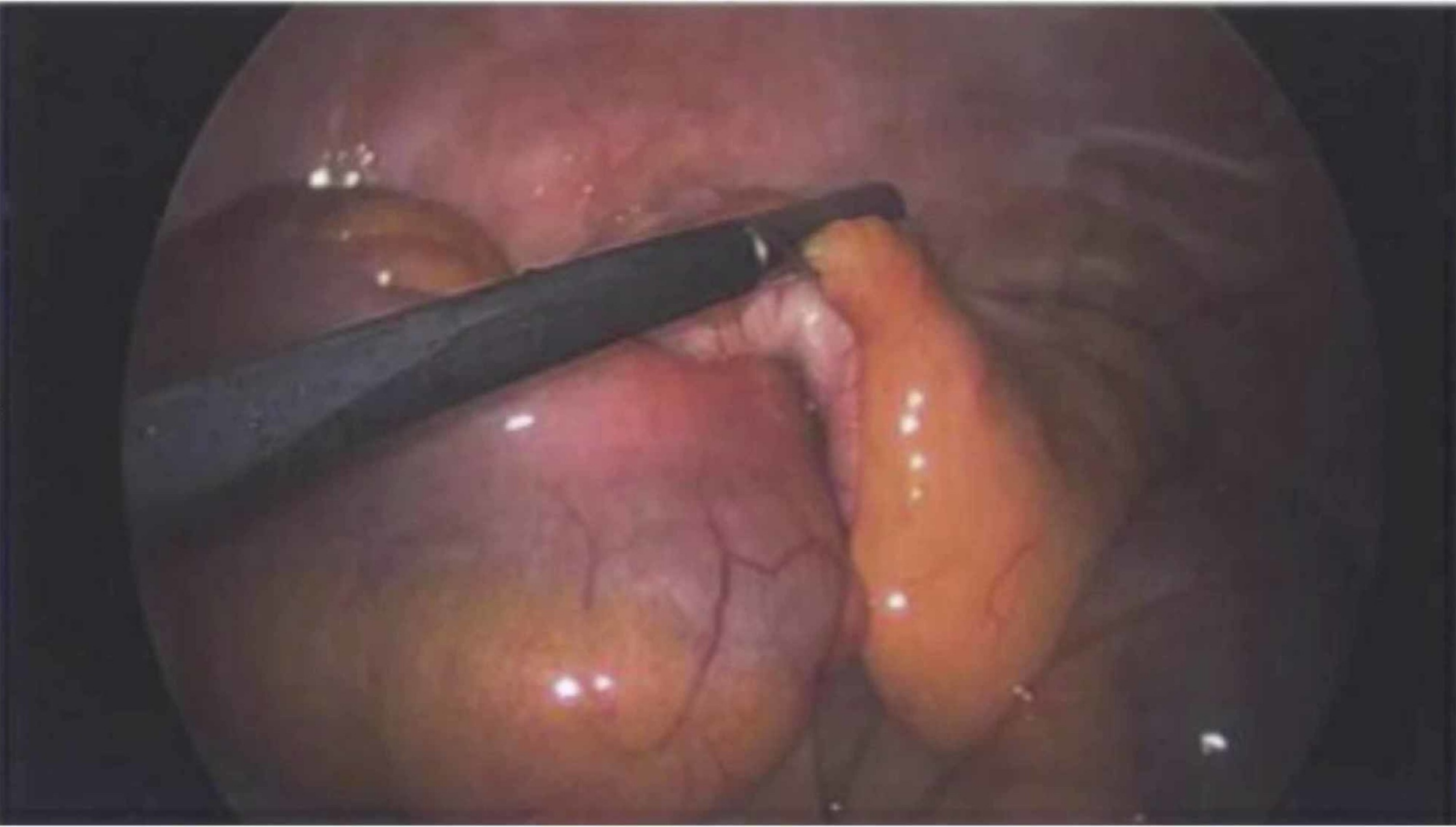
The ileocecal junction The junction of the ileum and the cecum is the confirmed location of the lead point of the intussusception. After the patient underwent a laparoscopic right hemicolectomy, the gross anatomical specimen was sent to pathology for further examination. This confirmed the presence of a submucosal leiomyoma with superficial mucosal erosion at and/or near the ileocecal valve.

A laparoscopic right hemicolectomy was planned. After trocar placement and gaining access to the abdomen, the inside of the right lower quadrant was visualized. It was noted that there was a firm intussusception at the ileocecal junction involving the terminal ileum. It was not manually reducible. The terminal ileum was mobilized, and a window was made in the mesentery. The ileum was divided using an Echelon 60-millimeter stapler (Echelon Corporation, California, US) at approximately 25 cm proximal to the ileocecal junction. The right colon and appendix were mobilized. The mesentery of the small bowel, cecum, and right colon was taken and ligated using the LigaSure (Medtronic plc, Minnesota, US). The colon was then transected using an Endo-GIA 60 load stapler (Medtronic) at the hepatic flexure. A side-to-side stapled anastomosis was then created. Hemostasis was ensured and the specimen was sent to the pathology department. The pathologist confirmed the presence of a submucosal leiomyoma with superficial mucosal erosion at and/or near the ileocecal valve (see Figure 3).

Over the course of the hospital stay, the patient developed leukocytosis and spiked fevers. Pain control was achieved with hydromorphone and transitioned to an acetaminophen and oxycodone combination and ibuprofen. She developed acute kidney injury (AKI) while in the hospital due to prerenal azotemia as a result of dehydration that resolved within two days. The patient’s Hb level dropped from 9.7 at admission to 6.6 g/dl. She was transfused one unit of packed red blood cells (RBCs), which helped to increase her Hb level. Over the next two days, the patient’s distension reduced, her nasogastric (NG) tube was removed, and she was started on a clear liquid diet. The diet was advanced to regular as she demonstrated tolerance for the clear liquid diet.

## Discussion

Our patient is a young 22-year-old adult who presented with intestinal obstruction secondary to intussusception caused by a benign leiomyoma. A leiomyoma is a benign tumor, which is also known as a fibroid.

Adult intussusception cases present over 70% of the time with symptoms of bowel obstruction, which are nausea, vomiting, and abdominal pain [2]. They less commonly present with melena, guaiac plus stool, fever, weight loss, constipation, diarrhea, or palpable abdominal mass [2].

Intussusception is a telescoping of the proximal segment of the bowel into the distal segment [2]. Because of that, intussusception can lead to bowel obstruction and later to intestinal ischemia because the blood supply of the affected bowel is compressed and thus compromised. Ischemia can lead to necrosis of the bowel segment, which can cause complications such as perforation and sepsis in patients [3-4]. Other potential complications that can occur are peritonitis and tumor seeding, which can occur as a complication of surgical intervention [4].

Only 1% of bowel obstruction cases is caused by adult patient intussusception [5]. Compared to pediatric cases of intussusception, adult patient cases will have a demonstrable etiology 70%-90% of the time and the most common cause is tumor [6]. Currently, most cases of intussusception involving the colon are due to malignant masses while most cases of intussusception of the small bowel are caused by benign masses [7]. For this particular case, a laparoscopic right hemicolectomy was performed because about 50% of intussusceptions are secondary to malignant lesions. Due to this, the best management is to resect the lesion without attempting to reduce the lesion [2].

Intussusception can occur in many different parts of the bowel. The incidence of intussusception in these locations varies in adult patients (see Table 1) [8].

**Table 1:** Incidence of different types of intussusception seen in adults

Location	Incidence (n=745) [8]
Enteroenteric	39%
Ileocolic	13%
Ileocecal	17%
Colocolic	17%
Appendiceal	4%

We were interested in seeing how our case compared to other reported cases of adult intussusception secondary to leiomyoma of the colon. We looked for cases where the patient was within the age group of 18-40 years of age. This was done because of the perception that intussusception occurs more commonly in adults older than 40 years and in pediatric patients [9]. Our search through PubMed yielded seven other reported cases similar to ours (see Table 2) [10-16].

**Table 2:** Cases of intussusception secondary to leiomyoma of the colon in adult patients There have been several cases of intussusception secondary to leiomyoma of the colon in adult patients. This table describes the name of the author, year, title of the study, age of the patient, gender, and location of the leiomyoma [10-16].

Year, Author	Name of Study	Age of Patient	Sex	Location of Leiomyoma
2012, Akbulut [10]	Intussusception due to inflammatory fibroid polyp: a case report and comprehensive literature review by Akbulut S.	38 years old	Female	Ileoileal
2015, Kimura et al. [11]	Adult intussusception secondary to inflammatory fibroid polyp	30 years old	Male	Ileoileal
2017, Mochizuki et al. [12]	Laparoscopic operation after pre-operative reduction of an intussusception-induced inflammatory fibroid polyp	35 years old	Female	Ileocecal
2017, Rais et al. [13]	An unusual cause of intestinal obstruction in a young adult patient: inflammatory fibroid polyp	22 years old	Male	Ileoileal
2013, Neishaboori et al. [14]	Jejunal intussusception caused by a huge Vanek's tumor: a case report	40 years old	Female	Jejunojejunal
2014, Patel et al. [15]	Jejunal intussusception: a rare cause of an acute abdomen in adults	30-year-old	Male	Jejunojejunal
1976, Mcgregor JC et al. [16]	Inflammatory fibroid polyp of the ileum—a rare cause of intussusception	38 years old	Female	Ileoileal

The earliest reported case of adult intussusception was published in 1976. The case described a 38-year-old female with an ileoileal intussusception secondary to a fibroid [16]. The case with the youngest aged patient was published in 2017 with a 22-year-old male patient with ileoileal intussusception secondary to a fibroid [13]. Of the seven reported cases found, 57% were ileoileal intussusceptions secondary to fibroids and 14% were ileocecal intussusception secondary to fibroids [10-16].

## Conclusions

Our patient had an ileocecal intussusception secondary to leiomyoma of the colon. She was symptomatic for bowel obstruction and treated with a laparoscopic right hemicolectomy. When examined further, it was discovered that there were reported cases similar to hers in academic literature. This case report indicates that intussusception, despite being rare in adult patients, should be considered a differential diagnosis when evaluating patients for abdominal pain.
